# Changes in eligibility for a subcutaneous cardioverter-defibrillator after implantation of a left ventricular assist device–A prospective analysis

**DOI:** 10.1371/journal.pone.0284419

**Published:** 2023-04-18

**Authors:** Christos Zormpas, Johanna Mueller-Leisse, Stephan Hohmann, Jörg Eiringhaus, Henrike Aenne Katrin Hillmann, Jan D. Schmitto, Christian Veltmann, David Duncker

**Affiliations:** 1 Department of Cardiology and Angiology, Hannover Heart Rhythm Center, Hannover Medical School, Hannover, Germany; 2 Department of Cardiac, Thoracic, Transplant and Vascular Surgery, Hannover Medical School, Hannover, Germany; 3 Center for Electrophysiology Bremen, Klinikum Links der Weser, Bremen, Germany; University Hospital *Paolo Giaccone*, ITALY

## Abstract

**Background:**

The number of left ventricular assist devices (LVADs) implanted in patients with end-stage heart failure is increasing. In this patient cohort, subcutaneous implantable cardioverter defibrillators (S-ICDs) could be a promising alternative to transvenous ICDs due to lower infection rates and avoidance of venous access. However, eligibility for the S-ICD depends on ECG features that may be influenced by an LVAD. The aim of the present study was a prospective evaluation of S-ICD eligibility before and after LVAD implantation.

**Methods:**

The study recruited all patients presenting at Hannover Medical School for LVAD implantation between 2016 and 2020. S-ICD eligibility was evaluated using the ECG-based and the device-based S-ICD screening test before and after LVAD implantation.

**Results:**

Twenty-two patients (57.3 ± 8.7 years of age, 95.5% male) were included in the analysis. The most common underlying diseases were dilated cardiomyopathy (n = 16, 72.7%) and ischemic cardiomyopathy (n = 5, 22.7%). Before LVAD implantation 16 patients were found eligible for the S-ICD according to both screening tests (72.7%), but only 7 patients were eligible after LVAD, 31.8%; p = 0.05). Oversensing due to electromagnetic interference was observed in 6 patients (66.6%) found ineligible for S-ICD after LVAD implantation. A lower S wave amplitude in leads I (p = 0.009), II (p = 0.006) and aVF (p = 0.006) before LVAD implantation was associated with higher rate of S-ICD ineligibility after LVAD implantation.

**Conclusion:**

LVAD implantation can impair S-ICD eligibility. Patients with lower S wave amplitude in leads I, II and aVF were more likely to be ineligible for S-ICD implantation after LVAD implantation. Thus, S-ICD therapy should be carefully considered in patients who are candidates for LVAD therapy.

## Introduction

An increasing number of patients with end-stage heart failure receives left ventricular assist devices (LVADs) to improve survival and quality of life [1,2]. However, LVAD therapy is associated with a high risk of systemic infections leading to significant morbidity and mortality [2,3]. The majority of patients undergoing LVAD implantation have an implantable cardioverter-defibrillator (ICD) [4], further increasing the risk of infection [5,6]. The subcutaneous implantable cardioverter-defibrillator (S-ICD) is a safe and effective alternative to the transvenous ICD that is associated with less systemic infections [7]. In contrast to the transvenous ICD using intracardiac signals for arrhythmia detection, the S-ICD records an ECG from one of three possible sensing vectors. To ensure appropriate arrhythmia discrimination by these ECG vectors, performance of an ECG-based S-ICD screening test is recommended before implantation [8,9].

Previous studies have shown that S-ICD eligibility in patients with LVAD is lower compared to patients with heart failure in general [5,10]. A novel device-based S-ICD screening tool was found to unravel potential device-device interference between LVAD and S-ICD [10,11]. Nevertheless, no data are available as to which extent the implantation of an LVAD itself impacts S-ICD eligibility, which is especially relevant for patients who are candidates for an LVAD.

The present study aimed to prospectively evaluate S-ICD eligibility in patients undergoing LVAD implantation. S-ICD eligibility was examined using the standard ECG based S-ICD screening test as wells as a novel device-based S-ICD screening test, previously described by our group [10].

## Methods

Patients presenting at Hannover Medical School for LVAD implantation were included in the study between September 2016 and October 2020 in a prospective non-randomized manner. After study inclusion, ECG-based and device-based S-ICD screening test were performed. Following successful LVAD implantation the ECG-based and device-based S-ICD screening test were repeated. Baseline parameters were retrieved from hospital records. A 12-lead ECG was recorded in accordance with international standards [12].

The study complied with the Declaration of Helsinki. The study was approved by the local ethics committee (3394–2016) and all participants provided written informed consent.

### S-ICD screening tests

S-ICD eligibility was evaluated using the ECG-based and the device-based screening test before and after LVAD implantation.

The ECG-based screening test requires special positioning of conventional limb electrodes according to manufacturer’s recommendations as previously described [5]. S-ICD eligibility was evaluated in both left and right parasternal position. In order to unravel potential device-device interference between S-ICD and LVAD, we also performed the device-based screening test. The device-based screening test uses a functional S-ICD generator connected to an S-ICD lead and fixed in the appropriate position with adhesive tape and contact gel as previously described [10]. This screening test aims to better imitate an implanted S-ICD system in situ and allows to test for correct sensing and potential electromagnetic interference. The device-based screening test was performed at simple as well as double gain setting of the S-ICD (Boston Scientific, Natick, MA, USA).

In the present study, S-ICD eligibility was defined as eligibility of at least one vector in left or right parasternal position in both supine and erect position.

### Statistical analysis

Continuous variables are presented as mean ± standard deviation. Differences among continuous variables were compared using unpaired t-test. Categorical variables are presented as numbers and percentages and were compared among subgroups using chi-square test. Values of p < 0.05 were considered statistically significant. Statistical analysis was conducted using SPSS software version 26 (IBM, Armonk, NY, USA).

## Results

Out of 46 patients evaluated for implantation of an LVAD at Hannover Medical School, 22 patients with S-ICD eligibility tests performed according to study protocol before and after LVAD implantation were included in the analysis. The remaining patients were excluded because LVAD implantation was either not performed (n = 7) or because no evaluation after LVAD implantation was possible due to death of progressive heart failure (n = 14), heart transplantation (n = 1) or patients lost to follow-up (n = 2). Baseline characteristics are shown in **Table 1**. **Table 2** summarizes the 12-lead ECG parameters recorded before LVAD implantation.

**Table 1:** Baseline patient characteristics (n = 22).

Parameter	n = 22
Age (years)	57.3 ± 8.7
Male (n, %)	21 (95.5)
Chest circumference (cm)	107.4 ± 11.9
Body mass index (kg/m^2^)	28.5 ± 6.8
Underlying cardiomyopathy (n, %)	
• Dilated cardiomyopathy	16 (72.7)
• Ischemic cardiomyopathy • Valvular cardiomyopathy	5 (22.7) 1 (4.5)
LVEF (%)	22 ± 6
Prior cardiac surgery (n, %)	14 (63.6)
LVAD type (n, %)	
• HVAD (Medtronic)	12 (54.5)
• HeartMate 3 (Abbott)	9 (41.0)
• HeartMate II (Abbott)	1 (4.5)
Minimal invasive operation technique (n, %)	12 (54.5)
Implanted ICD	21 (95.5)
Implanted CRT-D	10 (45.5)

LVAD = left ventricular assist device; ICD = implantable cardioverter-defibrillator.

**Table 2:** Electrocardiographic parameters (n = 22) before implantation of the left ventricular assist device.

Parameter	n = 22
Atrial rhythm (n, %)	
• Sinus rhythm	13 (59.1)
• Atrial fibrillation	8 (36.4)
• Atrial tachycardia	1 (4.5)
Heart rate (bpm)	79.2 ± 14.8
Cardiac axis (°)	-19.2 ± 89.2
QRS duration (ms)	136.0 ± 36.8
QRS morphology (n, %)	
• QRS duration < 120ms	10 (45.5)
• LBBB	1 (4.5)
• IVCD	2 (9.1)
• Paced	9 (40.9)

LBBB: Left bundle branch block; IVCD: Interventricular conduction delay.

### S-ICD screening before LVAD implantation

**Fig 1:**
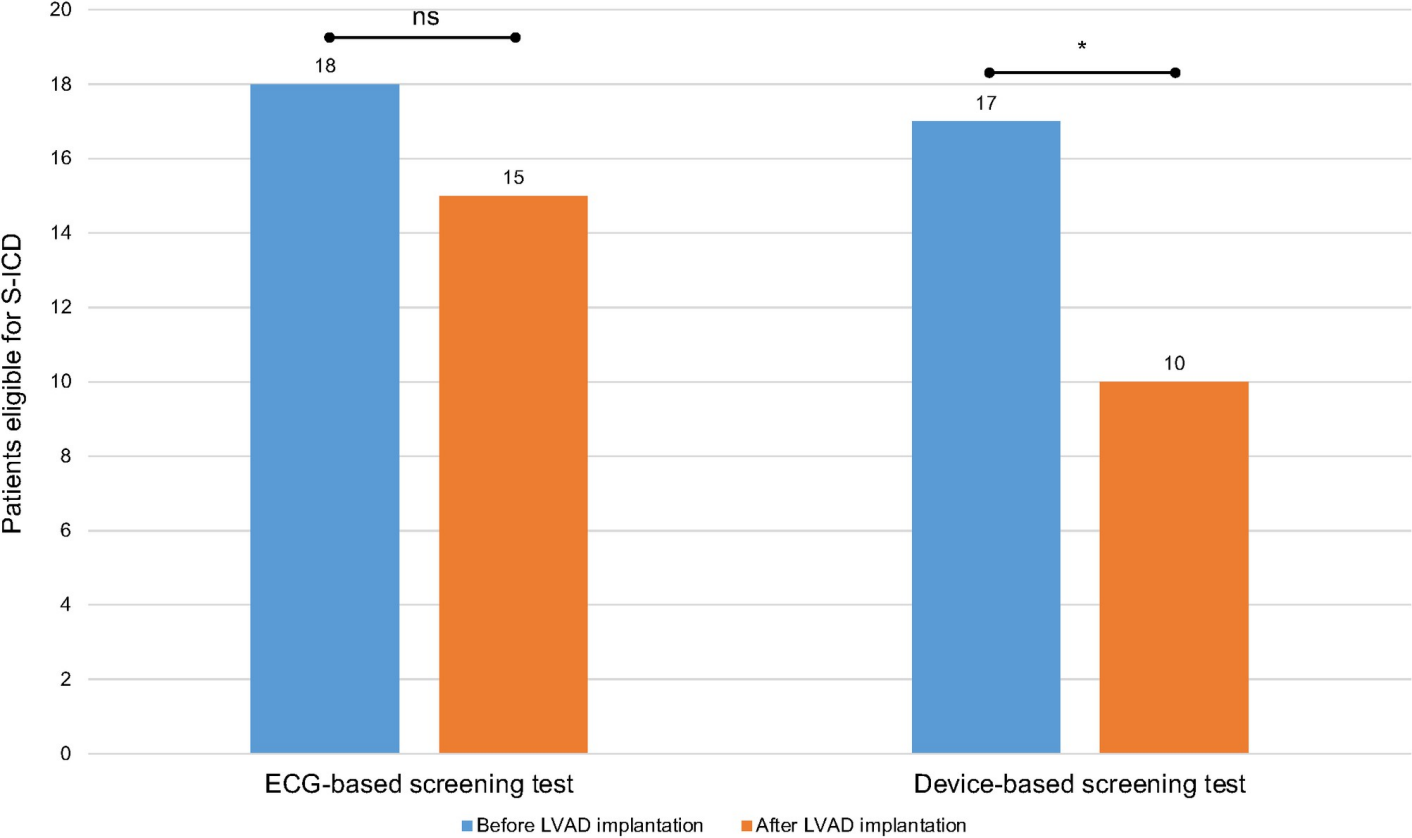
Number of patients found eligible for S-ICD implantation before and after LVAD implantation using the ECG-based screening test and the device-based screening test, respectively. LVAD = left ventricular assist device; S-ICD = subcutaneous implantable cardioverter-defibrillator; * = p < 0.05, ns = not significant.

According to the ECG-based screening test, 18 patients (81.8%) were found eligible for an S-ICD before LVAD implantation (**Fig 1**). Four patients (18.2%) had one eligible vector, 9 patients (40.9%) had two eligible vectors and 5 patients (22.7%) all three vectors eligible. Left parasternal position was favorable (18 patients with positive screening test) in comparison to right parasternal position (14 patients with positive screening test). Most common reason for screening failure was a high voltage QRS complex (57.6%) followed by a low voltage QRS complex (29.5%), T wave oversensing (11.9%) and oversensing of the following P wave (1%).

Using the device-based screening test, 17 patients (77.3%) had a positive screening test before LVAD implantation (**Fig 1**). Twelve patients (54.6%) had two eligible vectors and 5 patients (22.7%) all three vectors eligible. Left parasternal position was also favorable (9 patients with positive screening test) in comparison to right parasternal position (7 patients with positive screening test). Most common reason for screening failure was a low voltage QRS complex (99.2%) followed by T wave oversensing in only 0.8%.

### S-ICD screening after LVAD implantation

**Fig 2:**
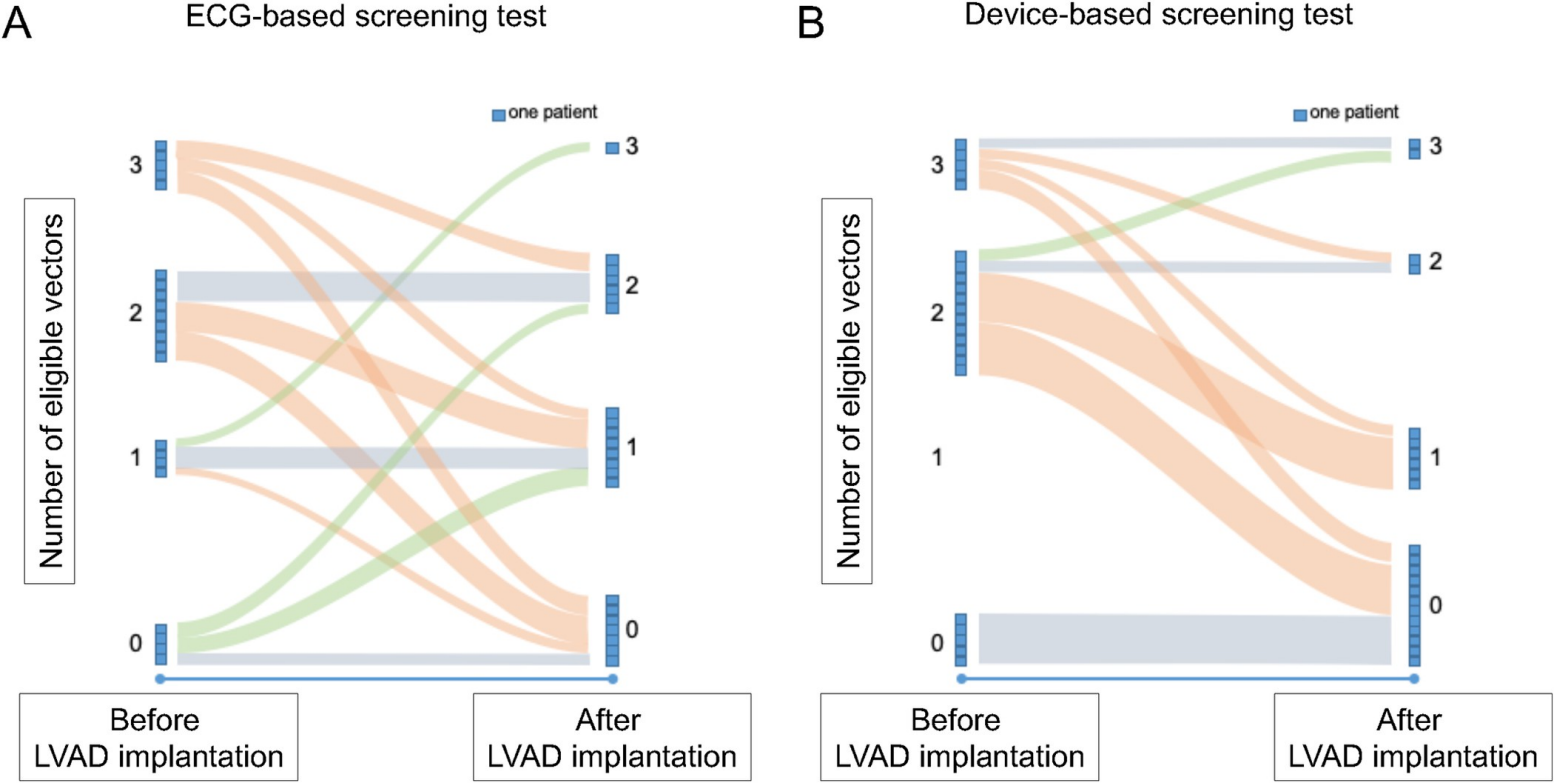
Sankey plot showing the number of eligible vectors before and after LVAD implantation using the ECG-based (A) and the device-based (B) S-ICD screening test. LVAD = left ventricular assist device.

Using the ECG-based screening test, 12 patients (54.5%) had less eligible vectors after LVAD implantation compared to the screening before LVAD implantation, while 6 patients had no changes in eligible vectors (27.3%) and 4 patients (18.2%) had more eligible vectors after LVAD implantation (**Fig 2**). Hence, 6 out of 18 patients with a positive test before LVAD implantation were found ineligible after LVAD implantation, and 3 out of 4 patients with a negative test before LVAD implantation showed a positive test afterwards, while 12 patients (54.5%) were found eligible before and after LVAD implantation. Taken together fewer patients were eligible found after LVAD implantation compared to the screening prior to LVAD implantation, without reaching statistical significance (before LVAD, n = 18, 81.8% vs after LVAD, n = 15, 68.2%; p = 0.75; **Fig 1**). Most common reason for screening failure according to the ECG-based test after LVAD implantation was a low voltage QRS complex (55.8%) followed by T wave oversensing (30.5%) and a high voltage QRS complex (11.5%). Oversensing of the following P wave was observed in 2.2%.

Using the device-based screening test, 14 patients (63.6%) had a reduction of eligible vectors after LVAD implantation, two patients had no difference in eligible vectors and one patient had an additional vector eligible after LVAD implantation (**Fig 2**). Hence, 7 out of 17 patients with a positive test before LVAD implantation were found ineligible after LVAD implantation. All patients with a negative test before LVAD implantation remained negative after LVAD implantation. Taken together, only 10 patients (45.5%) had a positive device-based screening test after LVAD implantation (before LVAD, n = 17, 77.3% vs after LVAD, n = 10, 45.5%; p = 0.02). Most common reason for screening failure was a low voltage QRS complex (80.8%) and oversensing due to electromagnetic interference (13%). T wave oversensing was observed in 6.2%.

### Eligibility for S-ICD implantation according to both tests

**Fig 3:**
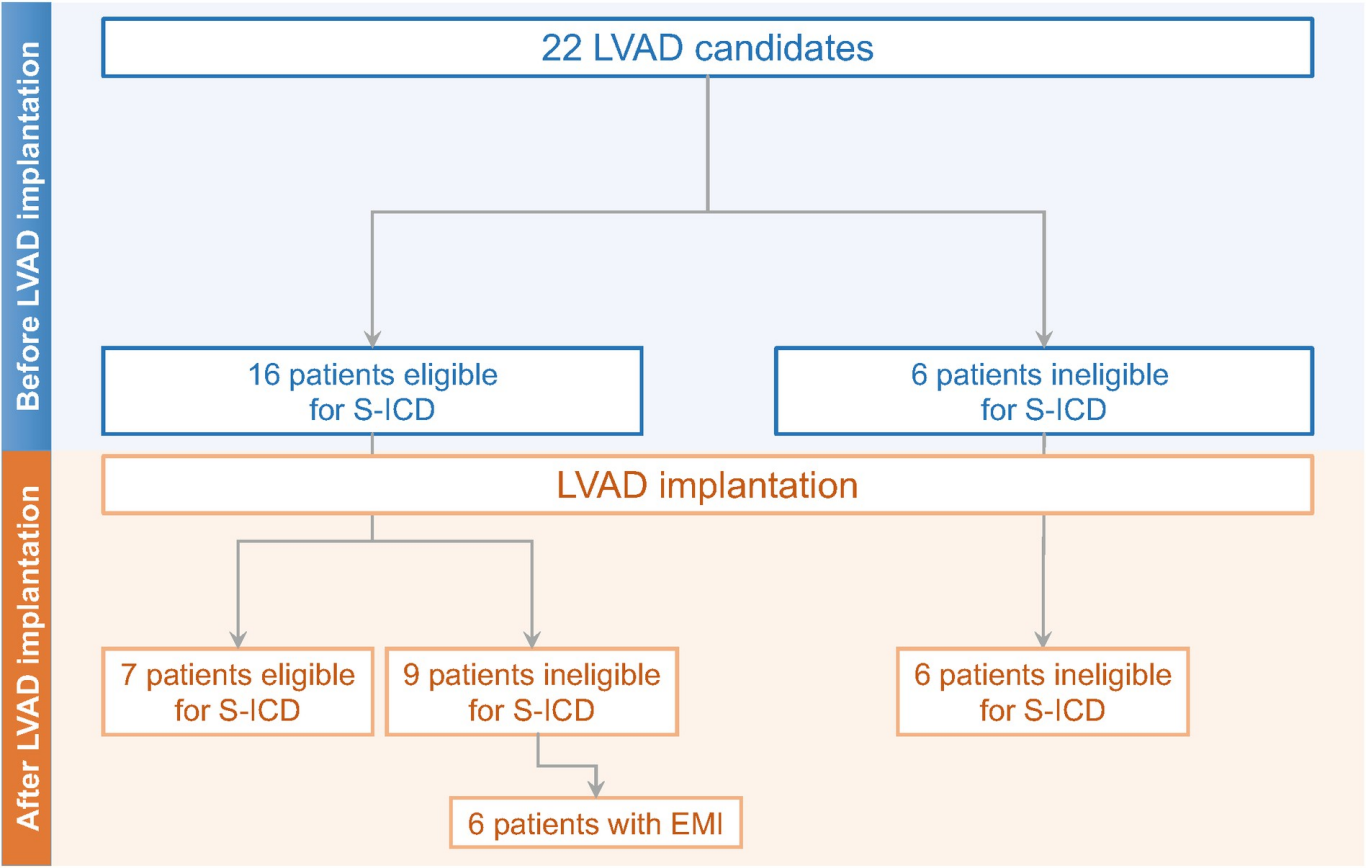
Evolution of S-ICD eligibility before and after LVAD implantation. LVAD = left ventricular assist device; S-ICD = subcutaneous implantable cardioverter-defibrillator; EMI = electromagnetic interference.

Taking both a positive ECG-based and a positive device-based S-ICD screening test for the same vector as a prerequisite for S-ICD eligibility, S-ICD eligibility was met in 16 individuals before LVAD implantation and in 7 patients after LVAD implantation (before LVAD, n = 16, 72.7% vs after LVAD, n = 7, 31.8%; p = 0.05). **Fig 3** demonstrates the eligibility rates before and after LVAD implantation. All patients found ineligible for S-ICD implantation before LVAD implantation (n = 6) remained ineligible after LVAD implantation.

In further analysis, a lower S wave amplitude in leads I (p = 0.009), II (p = 0.006) and aVF (p = 0.006) in the ECG before LVAD implantation was associated with S-ICD ineligibility after LVAD implantation. **Table 3** summarizes the baseline parameters according to S-ICD eligibility after LVAD implantation.

**Table 3:** Comparison of recorded parameters between patients eligible and ineligible for S-ICD implantation after implantation of left ventricular assist device.

Parameter	Eligible n = 7	Ineligible n = 15	p-value
Age (years)	52.9 ± 8.0	59.4 ± 8.6	0.901
Chest circumference (cm)	108.9 ± 17.0	106.7 ± 9.2	0.146
Body mass index (kg/m^2^)	31.0 ± 8.5	27.3 ± 5.9	0.391
Sinus rhythm (n, %)	4 (57.1)	9 (60.0)	0.899
Cardiac axis (°)	18.3 ± 108.2	-38.0 ± 75.5	0.353
QRS duration (ms)	136.6 ± 37.0	135.7 ± 38.1	0.501
Paced QRS complex (n, %)	2 (28.6)	7 (46.7)	0.421
Amplitude S wave in lead I (mV)	5.5 ± 13.0	0.9 ± 1.8	0.009
Amplitude S wave in lead II (mV)	16.2 ± 39.2	2.7 ± 3.8	0.006
Amplitude S wave in lead III (mV)	2.8 ± 4.2	5.6 ± 9.3	0.276
Amplitude S wave in lead aVF (mV)	16.7 ± 39.0	2.4 ± 3.4	0.006

## Discussion

The present study is the first prospective study evaluating S-ICD eligibility before and after LVAD implantation. Twenty-two patients undergoing LVAD implantation were examined regarding S-ICD eligibility using the ECG-based and the device-based S-ICD screening test.

The main findings of the present study are:

1. LVAD implantation led to a reduction in S-ICD eligibility.
2. The device-based screening test showed higher consistency compared to the ECG-based screening test and revealed oversensing due to electromagnetic interference in two thirds of patients found ineligible for S-ICD after LVAD implantation.
3. Patients with a lower S wave amplitude in leads I, II and aVF before LVAD implantation showed higher rates of S-ICD ineligibility after LVAD implantation.

The S-ICD is a promising alternative to the conventional transvenous ICD in patients with high risk for device-related infections, lead failures and altered venous access, such as patients with an LVAD [8,9,13]. Previous studies have raised concerns regarding device-device interference between LVADs and S-ICDs [14–16]. Previously published data have shown that S-ICD eligibility in patients with LVAD is lower than in patients without LVAD, with eligibility rates ranging from 40–73.3% [5,10]. Moreover, cases of inappropriate S-ICD shocks in LVAD patients have been reported [17–19]. These observations raise the question whether LVAD implantation itself negatively impacts S-ICD eligibility. In the present prospective study, we confirmed that S-ICD eligibility was lower after as compared to before LVAD implantation in a cohort of patients with end-stage heart failure.

An ECG-based S-ICD screening test performed before S-ICD implantation is the standard of care to ensure adequate S-ICD function [20]. However, a major concern in the context of LVAD and S-ICD coexistence is potential device-device interference, which can be unraveled by another, device-based S-ICD screening test that was previously described by our group. It had unmasked interferences in 14 out of 80 patients previously found eligible for an S-ICD with the ECG-based screening test [10]. These data suggest that both the ECG-based and the device-based screening test should be used in order to minimize device-device interference between LVAD and S-ICD. In the present study, for the first time we performed both screening tests in patients before as well as after LVAD implantation.

Regarding the ECG-based screening test only, the majority of patients (18 patients, 81.8%) was found eligible for S-ICD implantation before LVAD. This number is only slightly lower compared to previously observed S-ICD eligibility rates in heart failure patients without LVAD between 85.2–92% [21,22], the difference being possibly due to our exclusive end-stage heart failure cohort. After LVAD implantation, on the other hand, only 15 patients (68.2%) had a positive ECG-based screening test, in accordance with previously published data reporting S-ICD eligibility rates of 66.6–70% in patients with LVAD [5,10]. Interestingly, the ECG-based screening test showed quite inconsistent eligibility results, since 3 patients found ineligible before LVAD implantation unexpectedly showed a positive screening test after LVAD implantation.

According to the novel device-based screening test, on the other hand, all patients found ineligible before LVAD implantation remained ineligible after LVAD. Out of the 17 patients (72.3%) eligible before LVAD, 7 patients were no longer eligible after LVAD. This led to a significant reduction in S-ICD eligibility after LVAD by the device-based screening test (p = 0.02) and shows a higher consistency between test results using this method compared to the ECG-based screening. Only 10 patients (45.5%) had a positive screening test after LVAD according to the device-based test, and most patients found ineligible after LVAD implantation (6, 66.6%) showed oversensing due to electromagnetic interference. Electromagnetic interference is a known relevant issue with LVADs [11]. These data emphasize the importance of the device-based screening test in patients with an LVAD, and in patients who are potential candidates for an LVAD.

Taking both a positive ECG-based and a positive device-based S-ICD screening test for the same vector as a prerequisite for S-ICD eligibility, S-ICD eligibility was met in 16 individuals before LVAD implantation and only in 7 patients after LVAD implantation (before LVAD, n = 16, 72.7% vs after LVAD, n = 7, 31.8%; p = 0.05). Our results prove that LVAD implantation negatively impacts S-ICD eligibility.

Further analysis revealed that lower S wave amplitudes in leads I (p = 0.009), II (p = 0.006) and aVF (p = 0.006) in the twelve-lead ECG before LVAD implantation were associated with S-ICD ineligibility after LVAD implantation. Changes in these leads of the twelve-lead ECG have previously been described following LVAD implantation [23]. Such ECG features could serve as additional criteria when evaluating a patient who is a potential LVAD candidate for S-ICD eligibility.

### Strengths and limitations

Recruitment of patients in the present study was challenging since many patients were implanted with an LVAD in an acute setting and were not amenable for S-ICD screening before LVAD implantation. Moreover, 14 patients died before completion of the follow-up visit and could not be included in the analysis. These facts led to a rather small patient cohort limiting the analysis including multivariate analysis. However, the current study is the first evaluating S-ICD eligibility before and after LVAD implantation.

Performance of S-ICD screening test was not followed by actual S-ICD implantation. Consequently, it remains unclear if patients considered eligible for S-ICD implantation show adequate S-ICD sensing when implanted and vice versa. The device-based screening test is not established in clinical practice. Its sensitivity and specificity are not known and should be studied further, as our results suggest a better reliability compared to the standard test, and the potential to detect relevant device-device interferences.

## Conclusion

Implantation of an LVAD can impair eligibility for an S-ICD. Device-device interference can be challenging and the device-based S-ICD screening test could facilitate early detection of potential electromagnetic interferences. In patients with end-stage heart failure who are LVAD carriers or candidates for LVAD implantation, S-ICD therapy should be chosen carefully.
